# Guillain-Barré Syndrome Revealing Coexisting Cervical Spondylotic Myelopathy: A Diagnostic Pitfall

**DOI:** 10.7759/cureus.114887

**Published:** 2026-08-20

**Authors:** Hatim Boubekri, Anass Salah, Najoua Mankar Bennis, Saloua Khalfaoui, Ahmed Amine El Oumri

**Affiliations:** 1 Physical Medicine and Rehabilitation, Faculty of Medicine and Pharmacy, Mohammed I University, Oujda, MAR; 2 Physical Medicine and Rehabilitation, Mohammed VI University of Health Sciences, Rabat, MAR; 3 Physical Medicine and Rehabilitation, Mohammed V Military Instruction Hospital, Rabat, MAR

**Keywords:** atypical neurological evolution, cervical spondylotic myelopathy, diagnostic pitfall, guillain-barré syndrome, spinal cord compression

## Abstract

Guillain-Barré syndrome (GBS) is an acute inflammatory polyradiculoneuropathy characterized by rapidly progressive weakness and areflexia. Although the diagnosis is usually straightforward, atypical clinical evolution should prompt investigation for concomitant central nervous system pathology.

We report the case of a 64-year-old man initially diagnosed with severe GBS based on clinical presentation, cerebrospinal fluid (CSF) analysis, and electroneuromyography (EMNG) findings. Despite partial neurological improvement after intravenous immunoglobulin therapy, the patient later developed cervical pain, brisk reflexes, and a positive Babinski sign. Cervical magnetic resonance imaging (MRI) revealed severe multilevel cervical spondylotic myelopathy (CSM) with spinal cord compression and intramedullary T2 hyperintensity. Surgical decompression was subsequently indicated.

This case highlights the importance of reassessing patients with GBS who develop pyramidal signs or atypical neurological findings, as concomitant cervical myelopathy may be overlooked and delay appropriate management.

## Introduction

Guillain-Barré syndrome (GBS) is the most common cause of acute flaccid paralysis worldwide, characterized by symmetrical weakness of the limbs and hyporeflexia or areflexia, which reaches a maximum severity within four weeks [1]. The classic presentation of the syndrome does not typically pose a diagnostic challenge, but atypical variants are missed when not considered. To support diagnosis, polyradiculoneuropathy can be detected on nerve conduction studies, and cerebrospinal fluid (CSF) analysis can show albuminocytological dissociation, although both tests can be normal in the early stages [2].

Cervical spondylotic myelopathy (CSM) is the most common form of spinal cord injury in adults over 55 years of age [3]. Cervical spondylosis is a progressive disease defined by degenerative changes affecting the vertebrae, intervertebral disks, facets, and associated ligaments. These changes precipitate CSM through direct compression of the spinal cord and/or surrounding blood vessels [4]. Affected individuals may have subtle clinical findings, such as diminished hand dexterity or balance difficulties, or, in severe cases, symptoms of incontinence and complete paralysis [5].

This case highlights that an atypical course of GBS, especially when accompanied by the emergence of central neurological signs, should raise suspicion of an alternative underlying diagnosis and prompt further diagnostic investigation.

## Case presentation

A 64-year-old man with a medical history of type 2 diabetes mellitus and hypertension presented to the Emergency Department with acute-onset ascending weakness progressing to flaccid tetraplegia with generalized areflexia, associated with distal paresthesias. Initial laboratory investigations, including complete blood count and C-reactive protein, were unremarkable, and brain magnetic resonance imaging (MRI) showed no abnormalities. The patient’s condition rapidly deteriorated with the onset of respiratory failure and altered level of consciousness, requiring endotracheal intubation and admission to the Intensive Care Unit. CSF analysis revealed an elevated protein level (0.779 g/L) with a normal cell count (4 cells/mm³), consistent with albuminocytologic dissociation. Electroneuromyography (ENMG) demonstrated findings consistent with a demyelinating sensorimotor polyradiculoneuropathy. Based on clinical and paraclinical findings, a diagnosis of GBS was established. The patient was treated with intravenous immunoglobulins (30 g/day for five days), resulting in partial neurological improvement. The patient was successfully extubated after three weeks of mechanical ventilation.

Approximately two months after symptom onset, following this initial neurological improvement, muscle strength assessment using the Medical Research Council (MRC) scale showed persistent motor deficits, with muscle strength graded at 2/5 proximally and 1/5 distally in the upper limbs, and 2/5 in the lower limbs. During the rehabilitation phase, the patient developed cervical pain associated with severe neuropathic pain described as electric shock-like sensations. Neurological examination revealed generalized brisk deep tendon reflexes with a bilateral Babinski sign. No bladder or bowel dysfunction was present.

**Figure 1:**
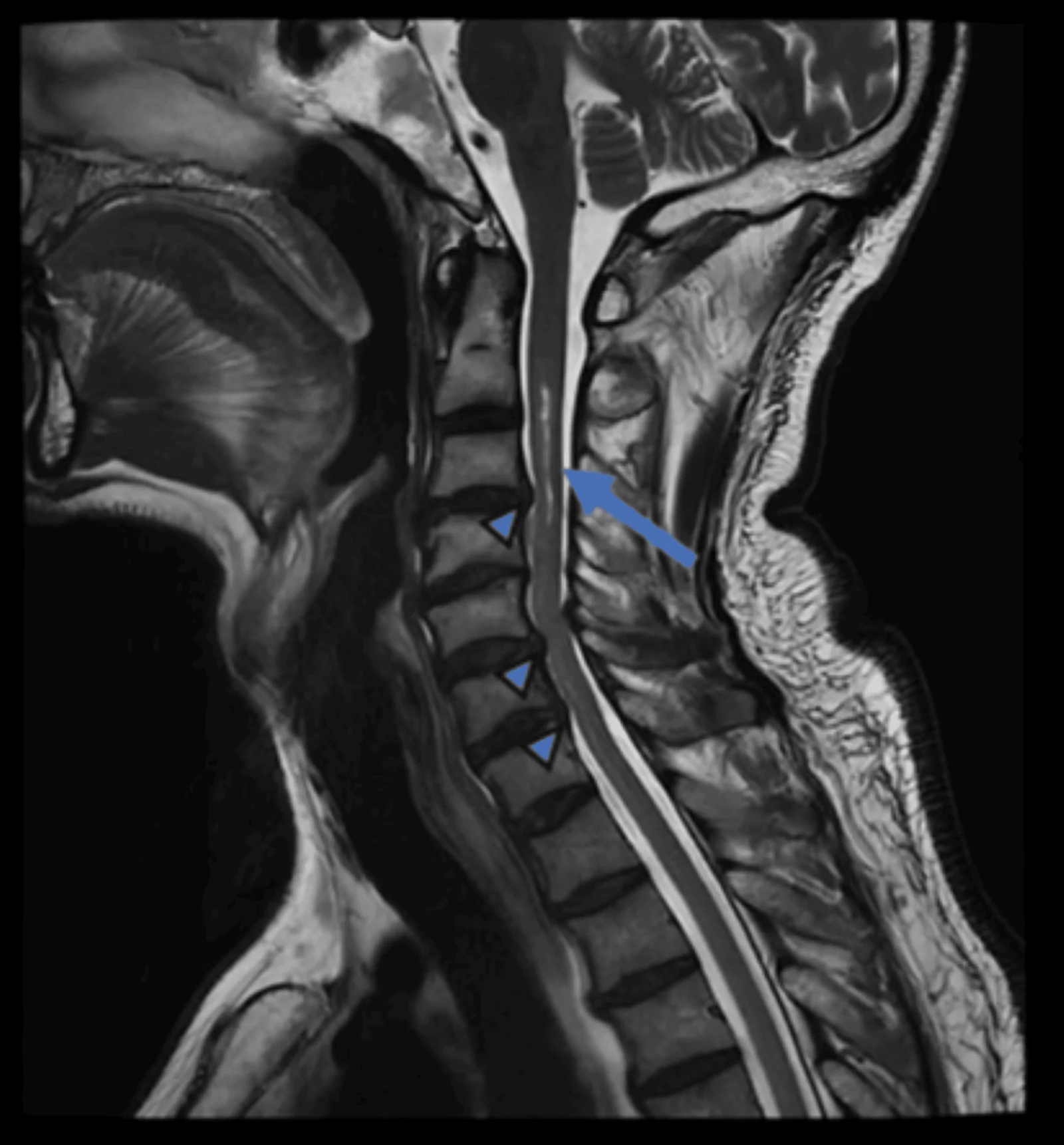
Sagittal T2-weighted MRI of the cervical spine Sagittal T2-weighted MRI of the cervical spine showing multilevel spinal cord compression from C3 to C7 (arrowheads). Central intramedullary T2 hyperintensity, extending from C2 to C5 (arrow). MRI: magnetic resonance imaging

**Figure 2:**
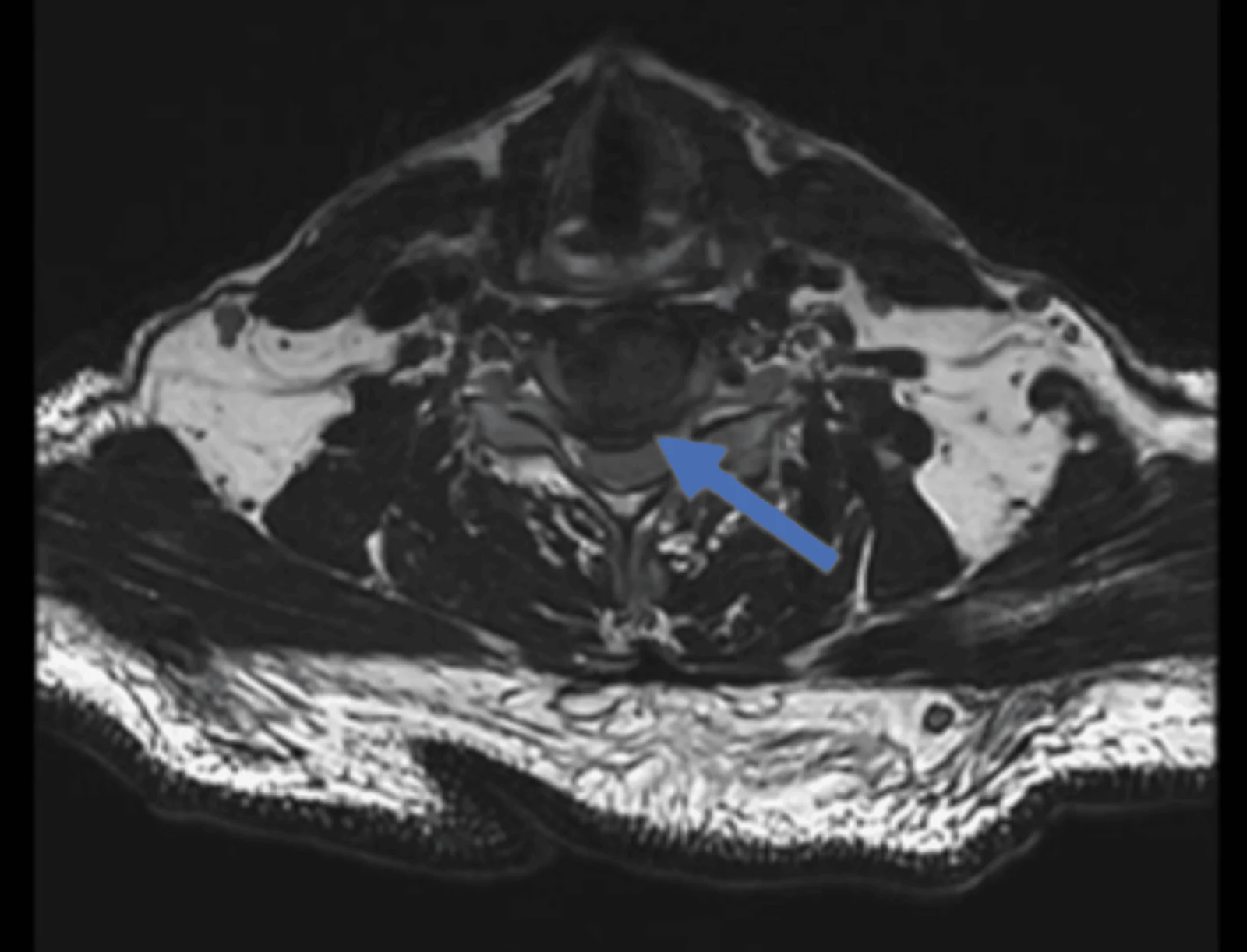
Axial T2-weighted MRI at the C5-C6 level Axial T2-weighted MRI at the C5-C6 level showing significant canal narrowing with marked spinal cord compression (arrow). MRI: magnetic resonance imaging

**Figure 3:**
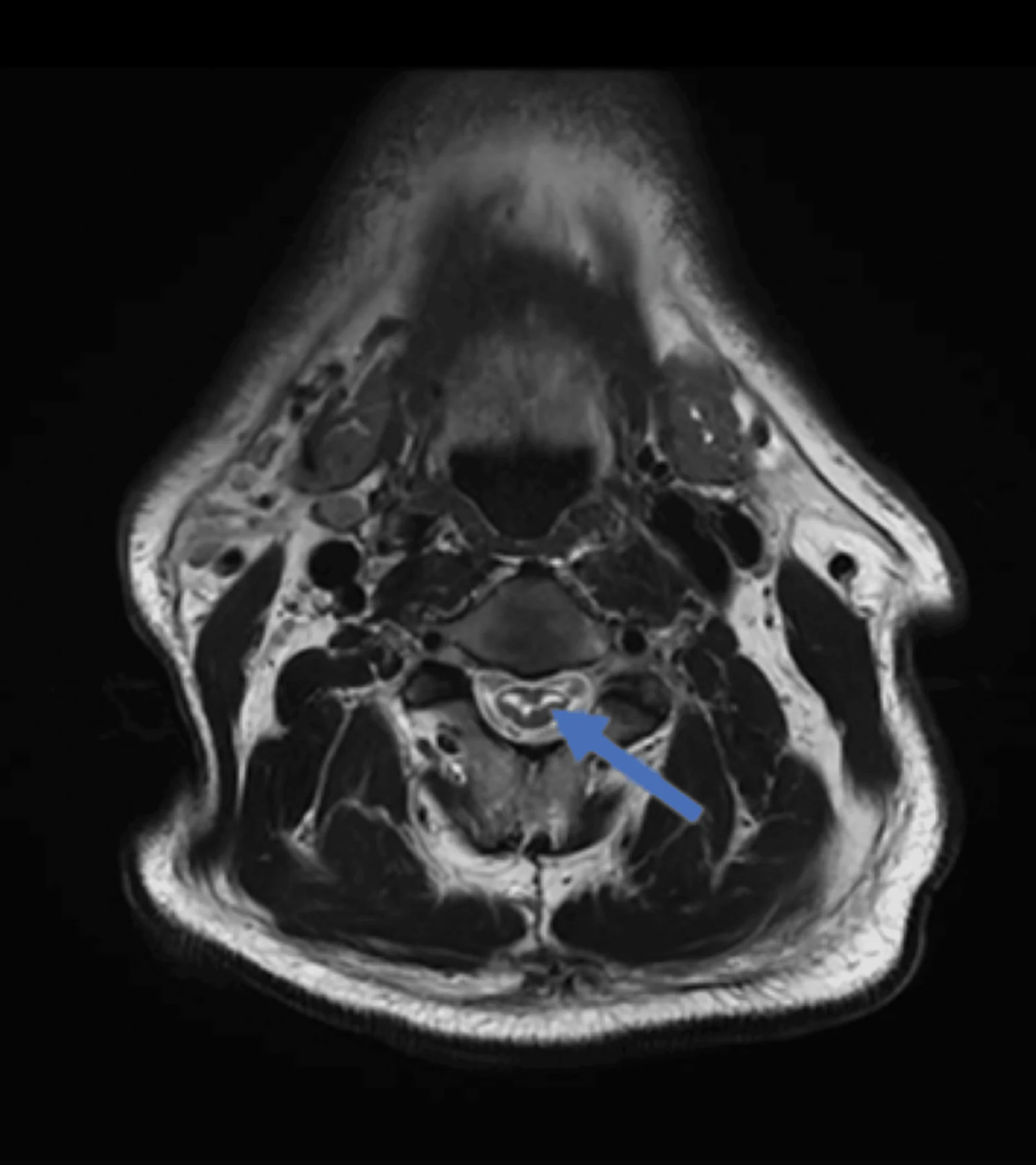
Axial T2-weighted MRI at the C2-C3 level Axial T2-weighted MRI at the C2-C3 level showing central intramedullary T2 hyperintensity (arrow), consistent with spinal cord signal changes. MRI: magnetic resonance imaging

Cervical spine MRI demonstrated multilevel CSM extending from C3 to C7 with associated spinal cord compression (Figure 1) and more marked narrowing at the C5-C6 level (Figure 2). Central intramedullary T2 hyperintensity extending from C2 to C5 was also observed, consistent with spinal cord signal changes (Figures 1-3). Posterior cervical laminectomy with spinal fusion was recommended. However, the patient declined surgical intervention and was subsequently transferred to another healthcare facility, precluding further follow-up.

## Discussion

GBS is a rare immune-mediated polyradiculoneuropathy. Patients typically develop rapidly progressive weakness and sensory deficits that can result in complete paralysis requiring mechanical ventilation. Diagnosis is based on clinical features, supported by CSF analysis and nerve conduction studies [6]. In this case, the initial diagnosis of GBS was supported by both clinical and paraclinical findings. The patient presented with acute flaccid tetraplegia and generalized areflexia, while CSF analysis showed albuminocytologic dissociation and electrophysiological (ENMG) findings supported the diagnosis of a demyelinating sensorimotor polyradiculoneuropathy. The clinical course was complicated by respiratory failure requiring mechanical ventilation, as seen in severe forms of GBS (25%-33% of cases) [7].

However, the patient’s subsequent evolution raised some concerns. The appearance of pyramidal signs, such as brisk reflexes and a positive Babinski sign, does not fit with a purely peripheral disorder and should alert the clinician. In GBS, reflexes are usually diminished or absent, so their return, especially in an exaggerated form, suggests possible involvement of the central nervous system [8]. This case highlights a common diagnostic trap: once a peripheral neuropathy is identified, it can be easy to attribute all symptoms to it and miss a coexisting central lesion. Persistent weakness alone may be explained by severe GBS. However, the appearance of generalized hyperreflexia, bilateral Babinski signs, cervical pain, or a sensory level should raise suspicion for concomitant cervical myelopathy, rather than incomplete recovery from GBS.

Although this combination is uncommon, similar cases have been increasingly described in the literature [9-11]. A recent pediatric case also showed how difficult it can be to recognize the coexistence of GBS and cervical myelopathy, as the overlap between peripheral and central features may delay the identification of an underlying spinal lesion [12]. In our patient, the cervical pain associated with significant neuropathic symptoms was more concerning and led to further tests. Cervical spine MRI showed multilevel CSM that explained the appearance of pyramidal signs. Cervical myelopathy is a documented source of increasing motor impairment and upper motor neuron symptoms [4]. Although causality cannot be established, pre-existing CSM may have contributed to the patient's persistent neurological deficits and delayed recovery.

From a practical perspective, this case highlights the need for continuous clinical reassessment in patients diagnosed with GBS. The emergence of pyramidal signs or other atypical features should prompt evaluation for central nervous system involvement, including spinal imaging when appropriate. Failure to identify a compressive spinal lesion may lead to delayed surgical intervention and poorer functional outcomes.

## Conclusions

This case reminds us that not all neurological worsening in GBS should be attributed to the disease itself. Paying attention to atypical features is key to identifying coexisting conditions and ensuring timely and appropriate management.
